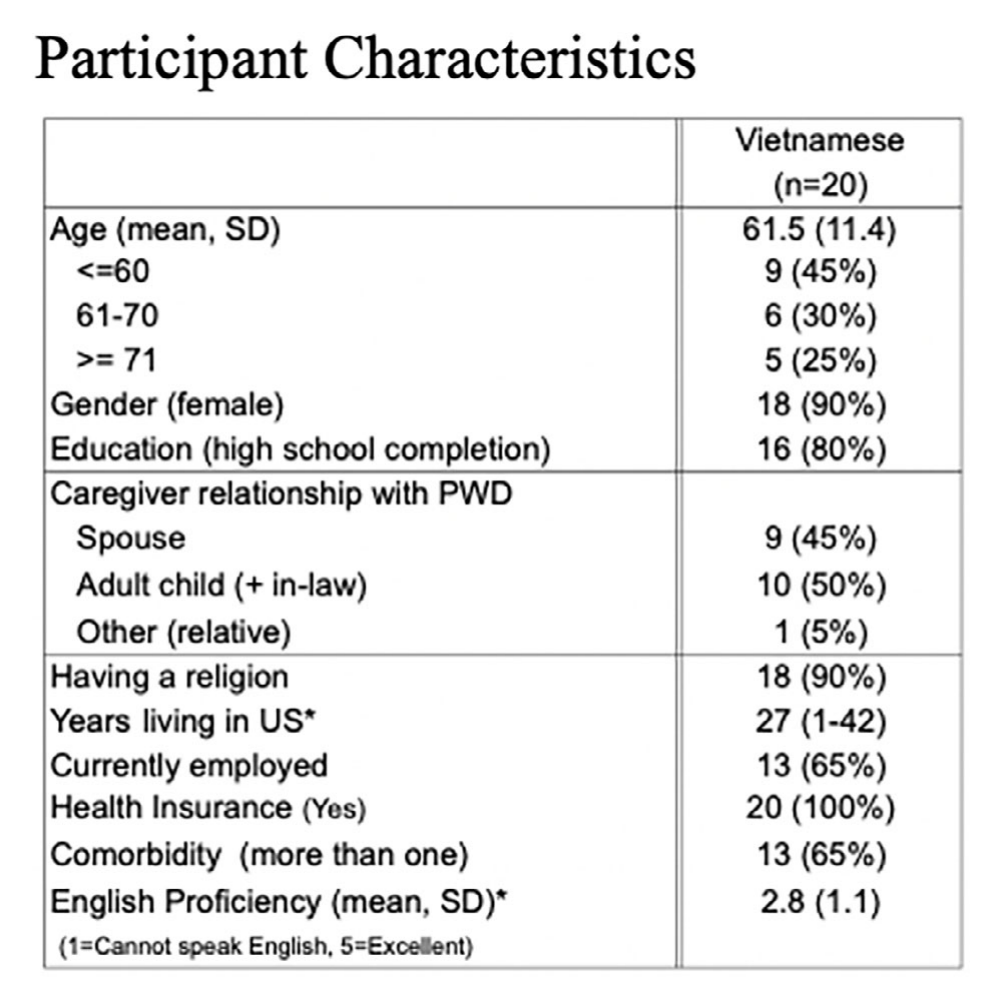# “I Lost My Freedom”: Exploring the Challenges of Caregiving for Vietnamese American Family Caregivers of Individuals with Dementia

**DOI:** 10.1002/alz70858_102550

**Published:** 2025-12-26

**Authors:** Hannah Nguyen, Michelle Zaragoza, Jung‐Ah Lee

**Affiliations:** ^1^ CALIFORNIA STATE UNIVERSITY, DOMINGUEZ HILLS, Carson, CA, USA; ^2^ UC Berkeley, Berkeley, CA, USA; ^3^ University of California, Irvine, Irvine, CA, USA

## Abstract

**Background:**

Family caregivers of individuals with dementia may get little respite due to around‐the‐clock care for a loved one. Over time, caregivers may experience the loss of personal freedom that negatively impacts their health and quality of life. Current research on dementia caregiving is limited in its inclusion of Asian American caregivers. This study explores the meaning of diminished freedom among Vietnamese Americans caring for a family member with dementia. The study's contributions deepen our understanding of interpersonal and sociocultural contexts that shape Vietnamese Americans caregivers’ sense of freedom and further guide culturally‐responsive interventions to promote caregiver wellness.

**Method:**

Twenty individual, semi‐structured interviews were conducted with Vietnamese American caregivers of a family member with dementia – recruited from ethnic specific agencies. Interviews were conducted in Vietnamese and simultaneously translated and transcribed into English. Our team coded coded data thematically using Dedoose.

**Result:**

Findings highlighted three dimensions to the gradual loss of freedom that parallelled the caregiving journey: physical, social, and psychological. Physical loss referred to caregivers’ inability to leave the house or have personal space due to around‐the‐clock care for loved ones. The loss of physical freedom was exacerbated by social losses, described as caregivers feeling isolated and disconnected from social support systems. Caregivers further highlighted their loss of psychological freedom – which arose from constant preoccupation with thoughts, worries, and emotions related to caregiving responsibilities. Caregivers juxtaposed the need for personal freedom with caregiving obligations, often resulting in a diminished sense of personal freedom as caregiving priorities take over. Caregiver expressed a desire to fulfill personal freedom while still upholding their familial duties and cultural values related to caregiving.

**Conclusion:**

Helping professionals who provide support to Vietnamese American caregivers should consider the physical, social, and psychological dimensions of freedom – embedded within sociocultural context – when developing culturally‐tailored interventions to promote caregiver wellness.